# Determinants of timing, adequacy and quality of antenatal care in Rwanda: a cross-sectional study using demographic and health surveys data

**DOI:** 10.1186/s12913-023-09231-y

**Published:** 2023-03-06

**Authors:** Gerard Uwimana, Mohamed Elhoumed, Mitslal Abrha Gebremedhin, Lin Nan, Lingxia Zeng

**Affiliations:** 1 Department of Epidemiology and Biostatistics, School of Public Health, Xi’an Jiaotong University Health Science Center, No 76 West Yanta Road, Xi’an, 710061 Shaanxi Province People’s Republic of China; 2 National Institute of Public Health Research (INRSP), BP. 695, Nouakchott, Mauritania; 3 Key Laboratory of Environment and Genes Related to Diseases, Xi’an Jiaotong University, Ministry of Education, Xi’an, 710061 Shaanxi P.R. China

**Keywords:** Antenatal care, Rwanda, Sustainable development goals

## Abstract

**Background:**

Antenatal care (ANC) is a recommended intervention to lessen maternal and neonatal mortality. The increased rate in ANC coverage in most Sub-Saharan African countries is not considerably reducing the maternal and neonatal mortality. This disconnection has raised concerns to study further the trend and determinants of the ANC timing and quality. We aimed to assess the determinants and trend of the timing, the adequacy and the quality of antenatal care in Rwanda.

**Method:**

A population-based cross-sectional study design. We used data from the 2010,2015 and 2020 Rwanda demographic and health surveys (RDHS). The study included 18,034 women aged 15–49 years. High quality ANC is when a woman had her first ANC visit within 3 months of pregnancy, had 4 or more ANC visits, received services components of ANC during the visits by a skilled provider. Bivariate analysis and multivariable logistic regression were used to assess the ANC (timing and adequacy), the quality of the content of ANC services and the associated factors.

**Results:**

The uptake of antenatal services increased in the last 15 years. For instance, the uptake of adequate ANC was 2219(36.16%), 2607(44.37%) and 2925(48.58%) respectively for 2010;2015 and 2020 RDHS. The uptake of high quality ANC from 205(3.48%) in 2010 through 510(9.47%) in 2015 to 779(14.99%) in 2020. Women with unwanted pregnancies were less likely to have timely first ANC (aOR:0.76;95%CI:0.68,0.85) compared to planned pregnancies, they were also less likely to achieve a high-quality ANC (aOR: 0.65;95%CI:0.51,0.82) compared to the planned pregnancies. Mothers with a secondary and higher education were 1.5 more likely to achieve a high-quality ANC (aOR:1.50;95%CI:1.15,1.96) compared to uneducated mothers. Increasing maternal age is associated with reduced odds of update of ANC component services (aOR:0.44;95%CI:0.25,0.77) for 40 years and above when referred to teen mothers).

**Conclusion:**

Low-educated mothers, advanced maternal age, and unintended pregnancies are the vulnerable groups that need to be targeted in order to improve ANC-related indicators. One of the credible measures to close the gap is to strengthen health education, promote family planning, and promote service utilization.

## Introduction

Antenatal care (ANC) describes the treatment provided to pregnant women and adolescent girls by qualified health-care professionals in order to maximize both mother and child's health outcomes throughout pregnancy [1]. Antenatal visits allow for the early detection and treatment of underlying conditions or pregnancy complications, and unfold to allow women to deliver in a health facility and to improve the use of emergency obstetric services [2, 3]. Despite numerous clinical and public health initiatives, Sub-Saharan Africa(SSA) continues to have the highest maternal mortality rates with recent estimates at 534 deaths per 100,000 live births [4], most deaths could be avoided if, skilled birth attendance and ANC visits were increased. A study conducted in 57 Low- and Middle- Income Countries (LMICs) revealed that both adequate and timing of first ANC were associated with reduced risks of neonatal mortality [5]. A study conducted in Kenya, a country in the same region of east Africa found that not attending any ANC was associated with neonatal mortality [6]. According to research, two-thirds of neonatal deaths could be avoided if all expectant mothers and newborns got specialized care throughout their pregnancies and deliveries [7–11].

Rwanda has been implementing the ANC policy adapted from the 2002 World Health Organization(WHO) recommendations, which recommended four ANC visits, the first visit taking place within the first trimester [12]. Rwanda's maternal death rate has dropped drastically in the recent two decades, from 1071 per 100,000 live births in 2000 to 249 per 100,000 in 2017 [4, 13]. The latter is still more than three times higher than the global maternal mortality objective set out in the Sustainable Development Goal(SDG) 3.1, which calls for a global maternal mortality rate of less than 70 per 100,000 live births by the year 2030 [14]. To achieve this purpose, the WHO recommend that every woman must complete timely and adequate ANC visits [14]. However, the most recent report showed that only 47% of the women in Rwanda had attained adequate ANC visits and that only 59% of the women had their first ANC in the first trimester of gestation [15]. In addition, a slight decline in maternal mortality has been observed in the two most recent RDHS [15, 16]. Women’s unenlightenment on pregnancy complications [17] and the perceived barriers to utilizing health care [18] were found to be hindering ANC attendance in Rwanda.

Antenatal visits alone do not promise high quality ANC. According to a recent study conducted in 91 low- and middle-income countries, one out of every three women who seek ANC do not obtain at least three antenatal services, such as blood pressure measures and blood tests [19]. There is little study on quality ANC, and the majority of ANC studies have focused on receiving recommended ANC visits [20–22]. A study conducted in Rwanda evaluated only the determinants of delayed ANC visits [23] and a recent study focused on investigating only on the perceived barriers to utilizing health care [18]. None of the studies assessed the impact of woman’s household decision making in the uptake, content and quality of ANC. Woman’s household decision making has been found to positively impact the uptake of ANC in SSA and LMICs [24–27].

This is the first study to comprehensively at national level examine the trends, the determinants of the timing, adequacy but also the quality of ANC considering the qualifications of the ANC providers in Rwanda.

## Methods

### Study design and data source

This study is a cross-sectional study using secondary data from three waves of Rwanda demographic and health survey (RDHS). The three waves include RDHS 2010, RDHS 2015 and RDHS 2020. The RDHS is a cross-sectional study using a stratified two-stage sampling design in which rural and urban place of residence are regarded as strata [15, 28, 29]**.** The census enumeration areas are considered as clusters and a full list of all households was later used as a sampling frame to choose which households should be interviewed. A nationally representative household sample is finally collected. The response rate has been high, above 99% for women across the three waves. The RDHS collects data on maternal and child health services covering a period within the preceding 5 years of the survey. Details on sampling design, sample size, study instruments, data collection, informed consent, and other associated procedures can be found elsewhere [15]. The RDHS data are accessible from the Measure DHS website at http://dhsprogram.com/data/available-datasets.cfm.

### Analytic sample

**Fig.1:**
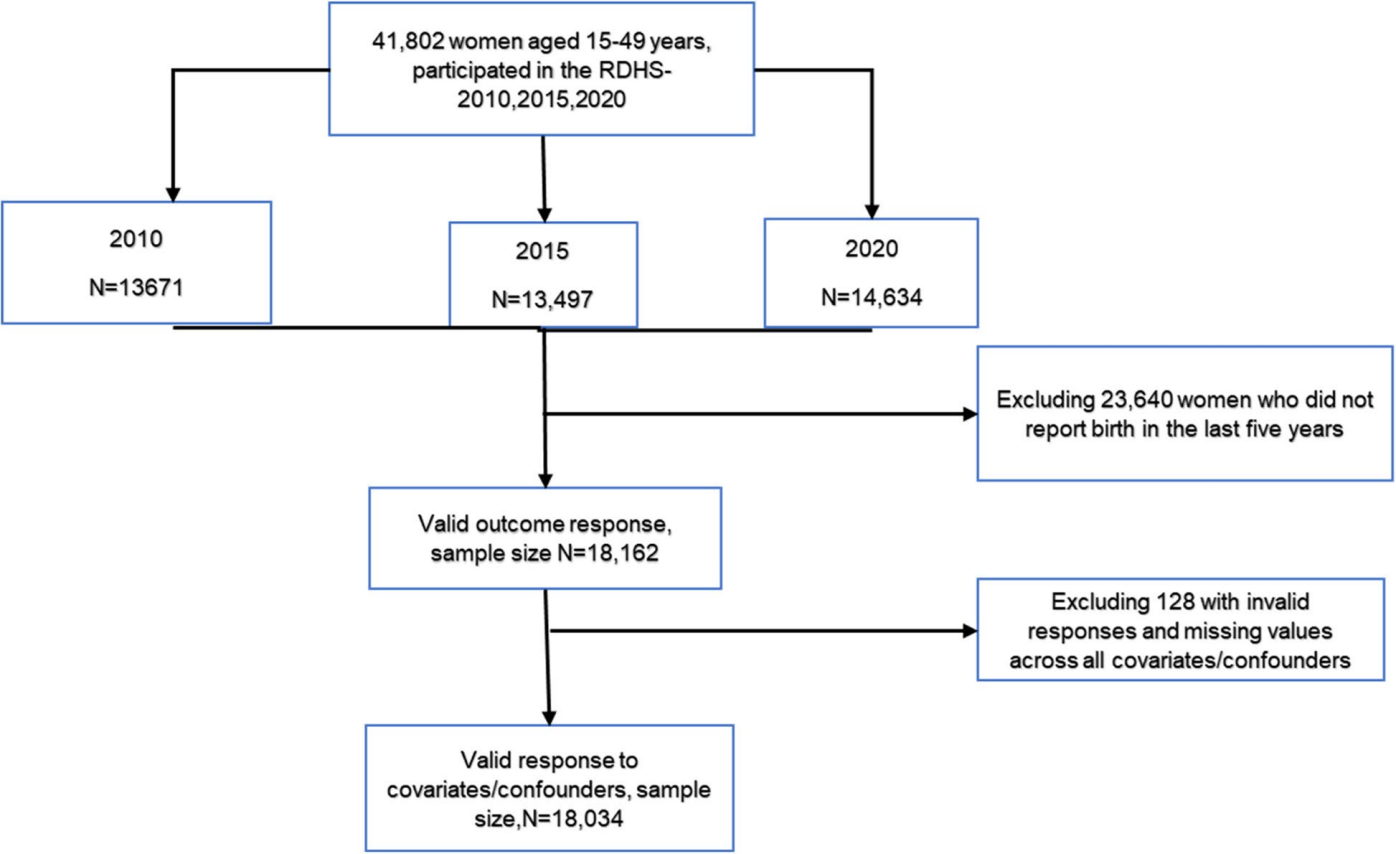
Flow chart of analytic sample selection

For the purpose of this study, the 2010, 2015, and 2020 RDHS individual recode (IR) datasets were merged based on established guidelines for managing DHS data. Women aged 15 to 49 years’ old who had a live birth in the five years prior to each survey and answered questions about ANC were included in this sample. Women with missing values or invalid responses to the key exposure, outcome, and possible confounders, such as “don’t know”, were removed. 18,034 of the 41,802 women who took part in the survey met the requirements for inclusion. More information is available in Fig. 1.

### Study variables

#### Outcome and exposure

The outcome variables of this study were (i) timing of first ANC visit; (ii) adequacy of ANC visits; (iii) services components of ANC; and (iv) High quality ANC (all quality indicators of ANC). ANC visits are crucial for preventing pregnancy-related issues, providing maternal and fetal health counseling, and preparing for birth in a health-care institution [30]. WHO recommends the first ANC visit should occur within the first trimester of gestation and at least four visits during the pregnancy. According to these guidelines, the outcome variables are dichotomous and are categorized as:(a) Timing of first ANC attendance (within 12 weeks of gestation = timely, beyond 12 weeks = delayed) and (b) adequacy of ANC attendance (frequency of 4 or more visits). There is no formal definition to help qualifying the (c) services components of ANC visits. For the purpose of analysis, the third outcome variable (c) was classified as either received or not received based on whether a woman had all five components of her ANC visits. This included receiving urine test, blood pressure measurement, blood sample test, tetanus injection, and iron and folic acid tablets. The choice to define this dependent variable this way is founded on the presumption that all the five components are crucial for quality pregnancy care [31]. The fourth outcome (d) High quality ANC is a composite of the first three and the receipt of ANC services by a skilled provider. A woman who had timely first ANC visit, had 4 or more ANC visits, received services components of ANC during the visits by a skilled provider was categorized as “yes” received high-quality ANC and “no” otherwise. A skilled ANC provider was considered as a medical doctor, a nurse or a midwife. The choice of this model was adapted from Bollini P and colleagues who proposed indicators to help measure the quality of ANC [32]; and referred to a recent study in India [33].

Various determinants of ANC utilization were examined as explanatory variables for their relevance in the uptake of ANC. These factors were adapted from Andersen’s behavioral model for healthcare use [34]. Many studies have made use of this model to investigate the determinants of antenatal care utilization [34–36]. These factors were: Age, type of place of residence (urban, rural), province, woman’s education level, employment status, wealth index, husband education level, husband employment status, access to media, involvement in health decision, birth order, place of antenatal care, perceived distance to health facility, the ease of getting money for treatment and child wantedness. Numerical values like age, birth order and years of education attended were grouped into categories. Women’s age in years was tabulated into groups (15–19 years, 20–24 years, 25–29 years, 30–34 years, 35 and above); birth order of the baby into four categories (1^st^,2^nd^,3^rd^,4^th^ and above); women’s and husbands’ education were classified as ‘no education’, ‘primary’, ‘secondary’ or ‘higher’ education. Access to media is a composite variable obtained from three variables (frequency of listening to radio/watching TV/reading newspapers) and is classified into not at all, less than once a week and at least once a week. The household wealth index was constructed using principal component analysis from items related to possession of durable assets, access to utilities and infrastructure, and housing characteristics. Each woman was ranked into five categories (poorest, poorer, middle, richer and richest) based on a household asset score, comprising 20% of the population [37, 38]. These five categories were later used to obtain three categories (poor, middle and rich).

### Statistical analyses

All the statistical analyses were conducted using Stata v14.0 [39]. Descriptive statistics for the sociodemographic characteristics of the study participants were generated by means of frequency and percentage as shown in Table 1.We used chi-square tests to identify demographic and socio-economic factors associated with each outcome variable. Crude odds ratios were generated by means of bivariate analyses to determine the odds of each outcome variable with explanatory variables. Potential factors with *p* < 0.20 [40, 41] in the bivariate analysis were retained for multivariate logistic regression in the final model. When covariates found to be collinear **(***r* >  = 0.8, using Pearson correlation test), the variable that was most correlated with the outcome variable of interest was retained. To account for clustering, stratification, and sample weight, we weighted all analyses using the survey module “*svyset*” stata commands.

**Table 1:** Sociodemographic characteristics of study participants

**Characteristic**	**Overall, weighted%**	**ANC within 12 weeks**	**ANC freq > = 4**	**Services components of ANC**	**High quality of ANC**
**Age at delivery**					
15–19	398(2.23)	187(46.8)	154(38.52)	58(20.58)	19(5.15)
20–24	3106(17.38)	1668(54.11)	1335(43.37)	538(22.94)	190(6.91)
25–29	4781(26.49)	2642(55.13)	2135(44.63)	1024(26.94)	450(10.41)
30–34	4378(24.05)	2305(52.17)	1877(42.27)	1060(27.53)	457(11.04)
35–39	3153(17.49)	1604(50.71)	1382(43.69)	665(23.38)	283(9.36)
40 +	2218(12.36)	958(42.54)	868(39.07)	276(13.06)	99(4.34)
***P-value***		< 0.001	< 0.001	< 0.001	< 0.001
**Mother education**					
no education	2576(14.59)	1110(43.07)	959(37.63)	365(15.77)	133(5.49)
primary	12,472(69.38)	6319(50.67)	5241(41.94)	2478(23.08)	993(8.47)
secondary and higher	2986(16.03)	1935(64.17)	1551(51.56)	778(36.53)	372(14.38)
***P-value***		< 0.001	< 0.001	< 0.001	< 0.001
**Mother employment**					
unemployed	3538(19.61)	1840(51.5)	1516(42.67)	765(26.56)	297(9.11)
employed	14,486(80.39)	7521(51.8)	6231(42.9)	2854(23.11)	1199(8.82)
***P-value***		0.791	0.832	0.0012	0.639
**Husband education**					
no education	2620(16.70)	1142(43.17)	1007(38.09)	411(17.27)	153(5.79)
primary	10,774(68.74)	5564(51.66)	4653(43.33)	2147(23.18)	905(9.06)
secondary and higher	2418(14.56)	1590(65.05)	1271(51.79)	615(32.54)	306(14.28)
***P-value***		< 0.001	< 0.001	< 0.001	< 0.001
**Husband employment**					
unemploye**d**	424(2.30)	237(59.81)	204(48.59)	126(34.04)	51(13.12)
employed	17,610(97.70)	9107(51.54)	7547(42.72)	3495(23.51)	1447(8.78)
***P-value***		0.0023	0.031	< 0.001	0.0049
**Married/partnered**					
no	3348(18.38)	1574(47.18)	1226(36.53)	592(22.15)	186(5.89)
yes	14,686(81.62)	7790(52.75)	6525(44.28)	3029(24.09)	1312(9.55)
***P-value***		< 0.001	< 0.001	0.053	< 0.001
**Religion**					
Christian	17,385(98.13)	9023(51.72)	7490(42.97)	3476(23.67)	1443(8.89)
Muslim	354(1.87)	198(55.03)	145(40.1)	89(29.01)	37(10.56)
***P-value***		0.258	0.329	0.063	0.294
**child wantedness**					
wanted then	10,606(58.85)	6117(57.46)	5052(47.48)	2217(25.4)	1035(10.73)
wanted later	4944(27.38)	2213(44.42)	1830(36.87)	1036(24.86)	335(7.06)
wanted no more	2481(13.78)	1032(41.71)	868(35)	367(15.64)	127(5.05)
***P-value***		< 0.001	< 0.001	< 0.001	< 0.001
**Getting money for treatment**					
big problem	9095(50.60)	4352(47.85)	3607(39.73)	1652(21.03)	614(7.26)
not a big problem	8938(49.40)	5011(55.69)	4144(46.06)	1969(26.73)	884(10.6)
***P-value***		< 0.001	< 0.001	< 0.001	< 0.001
**Perceived Distance to health facility**					
big problem	4307(24.30)	2045(47.31)	1683(39.13)	754(19.83)	300(7.41)
not a big problem	13,727(75.70)	7319(53.14)	6068(44.05)	2867(25.08)	1198(9.37)
***P-value***		< 0.001	< 0.001	< 0.001	< 0.001
**Involvement in healthcare decision**					
involved	11,659(79.29)	6285(53.58)	5233(44.69)	2475(24.82)	1075(9.84)
not involved	2984(20.71)	1487(49.7)	1275(42.8)	547(21.43)	235(8.53)
***P-value***		< 0.001	0.1	< 0.001	0.043
**Place of ANC**					
health center	16,526(92.26)	8433(50.94)	6911(41.79)	3337(23.85)	1350(8.76)
district hospital	546(2.97)	305(55.02)	256(46.23)	112(24.9)	47(8.99)
referral hospital	208(1.01)	144(66.25)	138(65.26)	45(27.57)	24(13.33)
health post/dispensary	435(2.28)	235(54.07)	218(49.67)	70(18.49)	37(9.08)
Clinic	277(1.47)	227(81.39)	206(74.82)	51(23.27)	35(14.09)
***P-value***		< 0.001	< 0.001	0.262	0.078
**Access to media**					
not at all	2641(14.76)	1341(50.66)	1062(40.1)	571(24.91)	229(9.26)
less than once a week	4191(23.19)	2022(48.44)	1678(40.05)	733(20.1)	260(6.48)
at least once a week	11,194(62.04)	5996(53.2)	5007(44.56)	2316(24.92)	1009(9.71)
***P-value***		< 0.001	< 0.001	< 0.001	< 0.001
**Birth order**					
1	4431(24.46)	2627(59.33)	2139(48.28)	636(21.3)	264(7.11)
2	3949(21.72)	2267(57.26)	1809(45.56)	1060(32.54)	480(13.06)
3	2987(16.58)	1568(52.47)	1289(43.15)	836(32.13)	356(12.65)
4 and above	6667(37.24)	2902(43.17)	2514(37.57)	1089(17.1)	398(6.01)
***P-value***		< 0.001	< 0.001	< 0.001	< 0.001
**Type of residence**					
urban	3481(15.78)	1980(54.89)	1611(45.74)	935(34.78)	382(11.62)
rural	14,553(84.22)	7384(51.13)	6140(42.31)	2686(21.92)	1116(8.39)
***P-value***		0.0052	0.0093	< 0.001	< 0.001
**Wealth index**					
poor	7833(43.58)	3785(48.45)	3140(40.07)	1413(20.91)	546(7.54)
middle	3445(19.65)	1797(52.24)	1466(42.53)	648(21.92)	264(8.22)
rich	6756(36.77)	3782(55.34)	3145(46.33)	1560(28.4)	688(10.89)
***P-value***		< 0.001	< 0.001	< 0.001	< 0.001
**Province**					
Kigali city	2109(11.83)	1043(48.97)	892(42.25)	573(36.26)	183(9.55)
south	4405(22.52)	2422(54.64)	2087(47.2)	917(24.76)	423(10.31)
west	4255(23.14)	2129(49.65)	1823(42.89)	754(21.29)	294(7.57)
north	2817(15.64)	1503(52.77)	1256(44.45)	694(29.38)	288(10.74)
east	4448(26.87)	2267(51.67)	1693(38.51)	683(17.24)	310(7.5)
***P-value***		0.0038	< 0.001	< 0.001	< 0.001
***Total***	*N* = 18,034	51.73%	42.85%	23.75%	8.88%

## Results

### Characteristics of study population

Table 1 summarizes a sample of 18,034 women aged 15–49. About 9159(51%) of the respondents were aged between 25 and 34 years, 14,486(80.39%) of the women were employed. A larger number of respondents 12,472(69.38%) had a primary level education and 11,194(62.04%) had access to media at least once a week. Rural habitants were 14,553(84.22%) and 7833(43.58%) were poor while the rich were 6756(36.77%). The percentage for married or partnered women is 14686(81.62%) and 11,659(79.29%) of respondents were involved in making decisions about healthcare. Mistimed and unwanted pregnancies was among 7425(41.15%) of the mothers.

Timely first ANC visit of the women was 9364(51.73%) whereas only 7751(42.85%) had adequate ANC visits. Women who received services components of ANC were 3621(23.75%) and only 1494(8.88%) achieved the high-quality ANC. A greater number of women 16,526(92.26%) had their ANC at a health center.

### Determinants of the timing of the first antenatal care visit

**Fig. 2:**
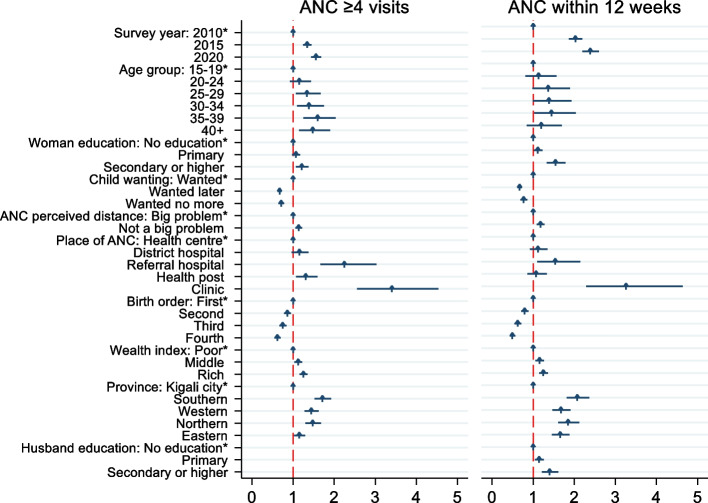
Factors associated with ANC frequency and timing. Adjusted odds ratios and 95%CI for the multivariate analysis between factors and frequency of ANC (on left) and timing of first ANC visit; *indicates reference category. ANC: antenatal care

Figure 2 shows that mothers who have secondary and higher education level had 50% more odds of timely first ANC visit (aOR:1.50;95% CI:1.29,1.74) compared to the uneducated mothers. Mothers who attended their ANC visit at a clinic had above three times the odds of timely first ANC visit (aOR:3.32;95%CI:2.32,4.75) compared to those who went to the health center to seek ANC services and those who had their ANC visit at a referral hospital had nearly 50% more odds of timely first ANC visit(aOR:1.52;95%CI:1.09,2.12) than those who had ANC visit at a health center. Rich mothers and those who perceived the distance to the health facility as not being a problem had above 10% more odds of timely first ANC visit (aOR: 1.20;95%CI:1.09,1.32& aOR: 1.16;95%CI:1.07,1.25) than poor mothers and those who perceived the distance to the health facility as a big problem respectively. Mothers whose husband’s had a secondary or higher education level had 37% more odds of timely first ANC visit (aOR: 1.37;95%CI:1.19,1.57) than the mothers whose husbands were uneducated.

Mothers who had mistimed and unwanted pregnancies had less odds of timing their first ANC visit (aOR:0.67;95%CI:0.62,0.73 and aOR:0.76;95%CI:0.68,0.85 respectively) compared to the mothers who wanted their pregnancy. Higher birth order of the child was negatively associated with the timing of the first ANC visit, and the higher the birth order, the less was the timing of the first ANC visit (aOR:0.63;95%CI:0.55,0.72 and aOR:0.50;95%CI:0.43,0.57 for 3^rd^ and 4^th^ or more respectively).

### Determinants of the adequacy of ANC visits

Figure 2 shows that early middle-age was positively associated with the adequacy of ANC when referred to teen mothers 15–19 years; aOR:1.67;95%CI:1.19,2.34 and aOR:1.57;95%CI:1.11,2.22 for women ages of 35–39 and 40 + years older. Mothers whose education level were secondary or higher had 19% more odds of having adequate ANC visits (aOR:1.19;95%CI:1.03,1.38) than uneducated mothers. Mothers who perceived the distance to health facility as not a problem had 13% more odds of going for 4 or more ANC visits (aOR:1.13;95%CI:1.05,1.23) compared to those who perceived the distance to health facility as a big problem. Rich women had 20% more odds of having adequate ANC visits (aOR: 1.20;95%CI:1.09,1.31) compared to the poor women. Women who went for ANC at a referral hospital had twice the odds of completing the recommended ANC visits (aOR:2.07;95%CI:1.51,2.83) compared to those who went at a health center, similarly the mothers who took their ANC visit at a clinic had 3 times the odds of completing the recommended ANC visits (aOR:3.38;95%CI:2.48,4.59) than those who went at the health center.

Mothers who mistimed and those who had unwanted pregnancies had less odds of completing 4 or more ANC visits (aOR:0.73;95%CI:0.67,0.79 and aOR:0.78;95%CI:0.70,0.87 respectively).

Birth order of the child was negatively associated with completing the recommended ANC visits (aOR:0.68;95%CI:0.60,0.77 and aOR:0.54;95%CI:0.48,0.62 for third and fourth order respectively), the higher the birth order of the child the less the odds of completing recommended ANC visits.

### Determinants of receiving services components of ANC

**Fig. 3:**
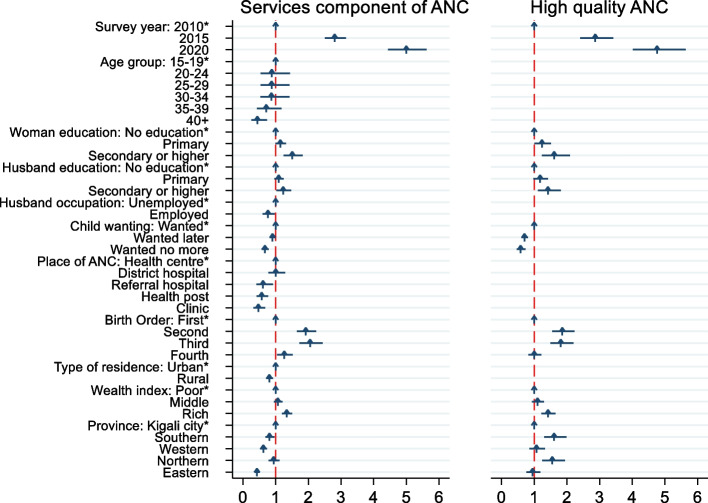
Factors associated with ANC service components and quality. Adjusted odds ratios and 95%CI between various factors and ANC service components and quality; *indicates reference category; ANC: antenatal care

Figure 3 shows that only 24.36% of the women who participated in the study got all services components of ANC. Maternal education increased the odds of receiving services component of ANC (aOR:1.44;95%CI:1.18,1.76). The increase in the husband’s education increased the odds of receiving services component of ANC for the woman. The odds of receiving services components of ANC by women whose husbands had secondary education level were 30% higher than that of women whose husbands did not have formal education (aOR:1.30;95%CI:1.07,1.57). Rich women had 18% more odds of receiving the services components of ANC (aOR:1.18;95%CI:1.04,1.34) than the poor women. Child unwantedness was found to be negatively associated with receiving services components of ANC services (aOR:0.67;95%CI:0.57,0.79). Older age was negatively associated with receiving services components of ANC (aOR:0.73;95%CI:0.42,1.27& aOR:0.44;95%CI:0.25,0.77) for 35 to 39 years old and 40 years and above respectively.

### Determinants of high-quality ANC

Maternal education was found to be positively associated with high-quality ANC. Mothers with secondary or higher education level had 50% more odds of receiving high-quality ANC (aOR:1.50;95%CI:1.15,1.96) than uneducated women. The husband’s education was found to be positively associated with receiving high-quality ANC (aOR:1.38;95%CI:1.07,1.77). Women in the rich tercile were positively associated with achieving high-quality ANC (aOR:1.26;95%CI:1.06,1.49). Women in southern (aOR:1.70;95%CI:1.36,2.14) and northern (aOR:1.60;95%CI:1.26,2.04) provinces had more odds of receiving high-quality ANC than women in other provinces in Rwanda. Mothers who mistimed (aOR:0.69;95%CI:0.59,0.79) and those with unwanted pregnancies(aOR:0.65;95%CI:0.51,0.82) had less odds of receiving high quality ANC.

**Fig. 4:**
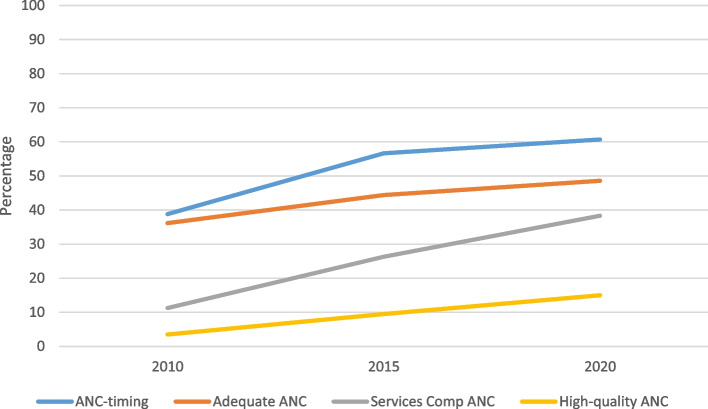
Trend of dependent variables by survey period

Figure 4 shows that all outcome indicator variables have been increasing over the survey period from 2010 to 2020. We found that only the timing of ANC was above 50% in 2015 and 2020. The high-quality ANC is very low.

**Fig. 5:**
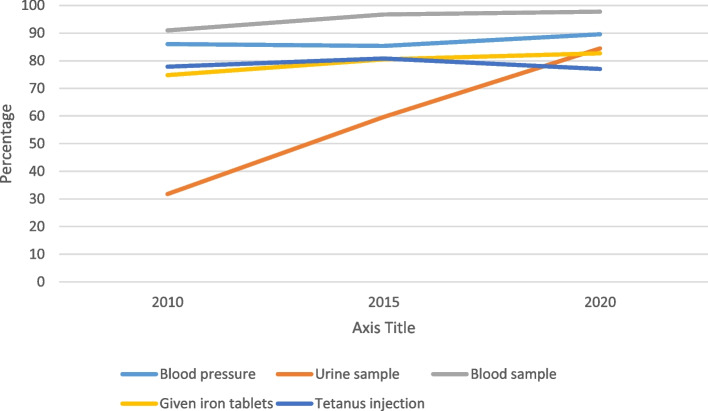
Trend of ANC services per survey year

Figure 5 shows that except for tetanus injection which did not steadily increase, there was an increase in ANC services provided over the survey years.

## Discussion

The current study investigated the factors of the timing of the first antenatal care visit, the adequacy of the antenatal care visits, the receipt of services components of ANC during the visits and the receipt of high-quality ANC in Rwanda. In general, higher maternal age is associated with timely and adequate ANC visits. This is in line with several other research that have demonstrated that maternal age is a predictor of maternal healthcare use [42–44]. Older age, on the other hand, was associated to insufficient ANC component services. Older women, who are more likely to have had previous pregnancies, may develop a sense of knowing what to look for and how to avoid difficulties, leading them to skip some medical testing. Another factor could be the women's dissatisfaction with past ANC visits. According to a recent study in Rwanda on person-centered Antenatal care (PCANC), a quarter of women felt disrespected during their ANC consultations [45]. When her dignity is threatened, an older woman is unlikely to complete the entire continuum of care [45–47].

Mothers who had secondary and above education had timely first ANC, adequate ANC visits, receive ANC components services and have a high-quality ANC compared to lower-level education mothers. Similar findings were observed in a community-based cross-sectional study conducted in Ethiopia in 2019, where maternal education was favorably associated with the timing, adequacy, ANC component services, and high-quality ANC [48]. Women with greater education have more autonomy and capacity to make educated and responsible health decisions [49, 50]. They have a greater knowledge of the information provided by health professionals to them and their children, including the necessity of maternity care continuity [49].

The wealth index was found to be positively associated with ANC component service timing, adequacy, and receipt, as well as high-quality ANC. Similar findings have been found in other African countries [48, 51]. In the Millennium Development Goals (MDGs) era, a previous study in Rwanda discovered that effective coverage of maternal and child health services was inequitable across socioeconomic status [52]. Rich women were more likely than poor women to obtain timely, appropriate, and high-quality ANC component services. This underutilization of ANC services by women in lower socioeconomic status highlights the need for health-care policymakers to design programs and provide incentives aimed at women in low-income households.

Mistimed and unwanted pregnancies had lower odds for timely, adequacy, receiving services component of ANC and high-quality ANC visits. Many studies got similar findings [18, 53, 54]. A study by Bahk J. et al. in Korea revealed that women’s sentiments on unplanned pregnancy might affect their health care seeking trends which can escalate the pregnancy complications [55]. High birth orders are negatively associated with timely and adequacy of ANC, and other studies got similar findings [56–58].This is assigned to the experience and awareness amassed over the ages on the appropriate behavior during pregnancy and the management of small complications for child’s higher birth orders. A cross-sectional study conducted in India argues that women tend to focus on their first pregnancy due to their inexperience [59]. This study found that, the 2nd and 3rd birth order are associated with high-quality ANC; This may result from a number of factors, among others the history of a risky pregnancy.

The odds of timely first visit and adequate ANC visits were higher in the southern, western and northern provinces than in Kigali city (the capital), this can be attributed to the recent decentralization of the Skilled Birth Attendant (SBA), to the health posts [14, 60, 61]. The lowest tier of healthcare facilities, health posts, offer ANC services to increase geographic accessibility to healthcare [61, 62]. A recent study showed the higher SBA utilization in most districts of the southern, western, and northern provinces [63].

The ease of access to a health facility was associated with timely and adequacy of ANC visits. Previous studies conducted in Rwanda showed that distance was an obstacle to adequate ANC visits and maternal health care utilization [18, 63]. Another study in Ghana showed that distance was a barrier to facility delivery [64].Women who have no problem of distance accessing the health facility do not encounter high travel costs, long time of travel and long waiting time to endure at a referral health facility [60].The increase in husband education was found to be positively associated with the timing of ANC, the receipt of services component of ANC and high-quality ANC received by the woman. Similar findings were found in Afghanistan were husband education was found to be positively associated with woman’s getting tetanus toxoid containing vaccine [65] and in Kenya, husband’s education increased the odds of the woman getting an ultrasound [51].This is explained by the increased awareness about mother and child health conditions acquired from the education of both the woman and husband, which impact the treatment-seeking behavior [66]. Residing in rural areas was found to be negatively associated with receiving ANC component services. Distance is a well-known stumbling block to healthcare consumption, as it is linked to a lack of transportation, limited access, and high expenses [67–69]. The risk of living in rural areas is compounded by the risk of distance. Other features of remoteness, such as inadequate road access, less communication between communities, disadvantaged socioeconomic status, adherence to cultural norms, and limited access to information, are captured when the two criteria are combined [68].

ANC Visits to the health post, referral hospital, and clinic were found to be negatively associated with receiving ANC component services. This demonstrates that the majority of women seek antenatal care at health centers, with the clinic and referral hospital being used only for specific tests like ultrasounds. According to a recent study conducted in Rwanda, the majority of nurses/midwives at health centers reported that ultrasounds were unavailable when they were needed [70].

Women with unwanted pregnancies and those with higher birth order of the child were less likely to receive services components of ANC services and high-quality ANC. This trend can be overcome with women empowerment; this will increase the utilization of latest birth control and control over the timing of pregnancy [71].

### Strengths and limitations

This is the first study to make a comprehensive assessment of the quality of ANC in Rwanda that accounts for not just the timing of the first visit and adequacy of ANC visits but also the ANC services delivered to the women and the qualification of the ANC providers. A major strength of the study is using a nationally representative population-based dataset with socio-economic variables on healthcare-seeking behavior. We excluded women who missed data across confounders, this might have produced a missing data bias. However, the bias is negligible since missingness is below 5%. It is possible that women may not have recognized if they received any of the services component of ANC but may have disclosed them because of social acceptability bias. The variable tracking the place of ANC also could not adequately account for potential variations in the health facility that women might have visited. To make the data easier to analyze, all the women who might have had ANC visits at different types of health facilities were grouped into one category. Similar to this, the ANC provider utilized in the analysis might not have been the one the women visited exclusively during the course of the pregnancy. The last stages of data collection took place during COVID-19 and the process had to stop for more than two months [15]. With the country in a total lockdown to contain the pandemic, women missed ANC visits and this might have slightly affected the findings. Findings related to inadequate, delays and lower quality ANC highlight the areas requiring health policy prioritization. This study did not include variables such as facility readiness, interpersonal relationships between providers and women, transportation and other cultural norms and beliefs that could have been determinants of ANC utilization. This study being cross-sectional could not determine cause and effect and were limited to the assessment of associations. However, the pooled analysis of three surveys may provide a robust estimate of those influencing factors. Further research would conduct a longitudinal study design to explore the risk factors of the timing, the adequacy and the quality of antenatal care in Rwanda.

## Conclusion

Although in the last 15 years, 2005–2020, Rwanda’s ANC indicators have improved significantly, however there is still a big gap with the SDG goal 3.2. To improve ANC-related indicators, the vulnerable groups that need to be focused on are low level educated mothers, advanced maternal age and unintended pregnancies. Targeting vulnerable groups, strengthening health education, family planning and promoting service utilization is one of the credible measures to bridge the gap. Among the four indicators, although the timeliness and frequency have improved significantly, the component of services provided and high-quality ANC are still very low. For the service provider: Enhancing the provision of necessary ANC services and the quality of the service is the key strategy.

## Data Availability

The datasets used during the current study are available from the DHS program website http://dhsprogram.com/data/available-datasets.cfm. Registration and application is required for access to data.

## Abbreviations

ANC: Antenatal Care
RDHS: Rwanda Demographic and Health Survey
WHO: World Health Organization
NISR: National Institute of Statistics of Rwanda
DHS: Demographic and Health Survey
SBA: Skilled Birth Attendance
MDGs: Millennium Development Goals
